# Mitochondrial‐targeted antioxidant ingestion acutely blunts *V*O_2max_ in physically inactive females

**DOI:** 10.14814/phy2.15871

**Published:** 2023-12-07

**Authors:** Ryan P. Hughes, Nicholas A. Carlini, Bradley S. Fleenor, Matthew P. Harber

**Affiliations:** ^1^ Clinical Exercise Physiology, Human Performance Laboratory Ball State University Muncie Indiana USA

**Keywords:** cardiorespiratory fitness, exercise, mitochondria

## Abstract

**Purpose:**

To determine the acute effects of a mitochondrial targeting antioxidant (MitoQ) on the metabolic response during exercise.

**Methods:**

Nine (*n* = 9) physically inactive females (age 47 ± 22 years) performed two trials (Placebo and MitoQ) in a double‐blind randomized cross‐over design. In both trials, participants performed an exercise protocol consisting of 3‐min stages at submaximal workloads followed by a ramp protocol to volitional exhaustion. Participants received either Placebo or MitoQ (80 mg) 1 h prior to exercise. Indirect calorimetry and cardiovascular measurements were collected throughout the duration of the exercise bout.

**Results:**

Submaximal metabolic and cardiovascular variables were not different between trials (*p* > 0.05). *V*O_2max_ was higher (*p* = 0.03) during Placebo (23.5 ± 5.7 mL kg min^−1^) compared to MitoQ (21.0 ± 6.6 mL kg min^−1^). Maximal ventilation was also higher (*p* = 0.02) in Placebo (82.4 ± 17.7 L/min) compared to MitoQ (75.0 ± 16.8 L/min). Maximal cardiovascular variables and blood lactate were not different between trials (*p* > 0.05).

**Conclusion:**

An acute dose of MitoQ blunted *V*O_2max_, which was primarily mediated by impairment of ventilatory function. These data suggest that the acute accumulation of exercise‐induced mitochondrial reactive oxygen species (mtROS) are necessary for maximal aerobic capacity. Further research is warranted on mtROS‐antioxidant cell signaling cascades, and how they relate to mitochondrial function during exercise.

## 1 INTRODUCTION

Maximal oxygen consumption (i.e., *V*O_2max_) was first described a century ago (Hill, 1924) and has since been extensively investigated for its relation to human performance in elite athletes and for health implications in the general and clinical populations (Ross et al., 2016). Defined as the highest rate that oxygen can be taken up and utilized by the body during intense exercise, *V*O_2max_ is an integrative variable that encompasses a multitude of body systems (Bassett Jr. & Howley, 2000). While *V*O_2max_ is generally considered to be primarily limited by oxygen delivery, mitochondrial function plays an important role in determining overall functional capacity (Bassett Jr. & Howley, 2000; Lundby et al., 2017). Specifically, mitochondrial efficiency is directly related to *V*O_2max_ as mitochondrial function is correlated with overall oxygen utilization capabilities (Blomstrand et al., 1997). Resultingly, mitochondrial function is crucial for both submaximal and maximal exercise performance alike (Bassett Jr. & Howley, 2000; Blomstrand et al., 1997; Hargreaves & Spriet, 2020; van der Zwaard et al., 1985).

Reactive oxygen species (mtROS) are oxygen derived non‐radical and radical species that originate in the mitochondria and can acutely alter cellular function in a multitude of ways (Goodpaster & Sparks, 2017; Mittler, 2017). The endogenous antioxidant system neutralizes mtROS produced by mitochondria and optimal cellular function is dependent on this balance. mtROS increase dramatically during acute exercise and may induce oxidative stress when mtROS accumulation exceeds antioxidant function, which has been shown to lower exercise capacity by hindering mitochondrial function thus causing inefficient substrate utilization (Louzada et al., 2020; Palmer & Clegg, 2022; Sies & Jones, 2020). Further, physically inactive individuals demonstrate higher levels of oxidative stress at rest and during exercise compared to those who regularly exercise (Goodpaster & Sparks, 2017; Rossman et al., 2018; Rynders et al., 2018). Chronic oxidative stress is associated with the pathogenesis of a variety of metabolic and cardiovascular disease states (Incalza et al., 2018). Therefore, maintaining the mtROS‐antioxidant balance is essential for preventing the development of chronic diseases.

Mitoquinol mesylate (MitoQ) is a commercially available supplement that consists of a ubiquinone moiety attached to a lipophilic cation, thus making it a mitochondrial targeted version of coenzyme Q10 (Murphy & Smith, 2007). MitoQ accumulates within the inner mitochondrial membrane where it acts to reduce mtROS produced directly from the electron transport chain and is a recyclable antioxidant allowing for sustained activity within the mitochondria. Therefore, it may be favored over traditional nonspecific antioxidants such as vitamins C and E, which cannot surpass the phospholipid bilayer of the mitochondria, and instead accumulate outside of it (Broome et al., 2021; Pham et al., 2020; Williamson et al., 2020). Preclinical and recently published clinical findings demonstrate the benefits associated with oral MitoQ supplementation for ameliorating oxidative stress (Rodriguez‐Cuenca et al., 2010; Rossman et al., 2018). However, the influence of MitoQ during exercise in humans remains unclear (Broome et al., 2021, 2022; Masoumi‐Ardakani et al., 2022; Park et al., 2020; Williamson et al., 2020). Previous studies have analyzed a variety of dosages and population groups, yet none have investigated the acute effects of MitoQ on metabolic regulation during both submaximal and maximal exercise in apparently healthy but inactive adults. Thus, the primary aim of this study was to examine if MitoQ alters the metabolic responses during submaximal and maximal exercise. Our hypothesis was that MitoQ supplementation will acutely enhance mitochondrial function, thus improving submaximal substrate utilization and maximal oxygen utilization capabilities.

## 2 METHODS

### 2.1 Study design

**FIGURE 1:**
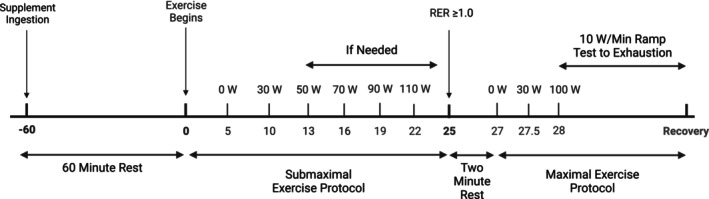
Timeline of experimental trials. Participants completed two trial days in a randomized double‐blind, placebo‐controlled crossover design. Timeline in minutes. RER, respiratory exchange ratio.

This study used a double‐blind, placebo‐controlled crossover design to examine the acute effects of MitoQ supplementation on metabolic responses during exercise (Figure 1) and was registered at clinicaltrials.gov as NCT06069245. Participants visited the Clinical Exercise Physiology (CEP) Laboratory within the Ball State University Human Performance Laboratory three separate times. Participants were instructed to fast from food and drink (other than water) for a minimum of 12 h prior to each visit and abstain from vigorous exercise for 24 h prior to each visit. Participants were also required to stop any other antioxidant supplementation and refrain from taking nonsteroidal anti‐inflammatory drugs (NSAIDs) for 72 h prior to each trial. In addition, participants were asked to consume the same meal of their choosing at the same time on the night before each lab visit and were provided a snack (Ensure) to consume 12 h prior to the time of the trial to further standardize dietary intake prior to each trial. Females still menstruating completed both visits during the early follicular phase.

During the first visit, written informed consent was collected and a health history questionnaire was completed to confirm eligibility. The first visit also included the collection of baseline resting data including anthropometrics, body mass index (BMI and DXA), resting ECG, glucose and lipid panel, and assessment of vascular hemodynamics. Upon arriving at the CEP lab for visits two and three, height and weight were measured, followed by consumption of either 80 mg of MitoQ (MitoQ Limited, Auckland, NZ) or placebo which consisted of empty gelatin capsules similar to our previous research (Harber et al., 2021). Participants were given 15 min to consume the capsules with water ad libitum. Visits were separated by a 1 week washout period to ensure that individuals had no circulating MitoQ and recovery from the exhaustive exercise protocol (Park et al., 2020; Rossman et al., 2018).

### 2.2 Participants

A sample of nine physically inactive females (age 47 ± 22 years; BMI 28.5 ± 3.8 kg/m^2^; four premenopausal) completed all study procedures. Resting physiological descriptors can be viewed at the 0 W workload timepoint in Table 1. Participant recruitment was not restricted to females; however, the only eligible individuals that completed all procedures were females. Two female participants did not meet the study criteria and one male participant did not have a fully complete data set to be included in data analysis. Participants were required to be physically inactive defined as participation in <150 min per week of moderate physical activity or < 75 min per week of vigorous activity. Individuals with a BMI greater than 35 kg/m^2^, Type II diabetes, current smokers, and individuals prescribed a beta blocker were excluded from the study. A resting blood pressure (BP) greater than 160/100 mmHg was also chosen as an exclusion criteria based off the Joint National Committee on Prevention, Detection, Evaluation, and Treatment of High Blood Pressure classification for Stage II hypertension (Chobanian et al., 2003). Individuals with signs or symptoms suggestive of cardiovascular or metabolic disease were also excluded.

**TABLE 1:** Individual workload submaximal exercise responses during Placebo and MitoQ trials.

Variable	Trial	0 W	30 W	50 W	*p*‐value (time)	*p*‐value (group)	*p*‐value (interaction)
VCO_2_ (L/min)	Placebo	0.22 ± 0.04	0.58 ± 0.05	0.81 ± 0.07	<0.0001	0.45	0.33
	MitoQ	0.22 ± 0.05	0.57 ± 0.08	0.77 ± 0.08			
RER	Placebo	0.85 ± 0.05	0.83 ± 0.05	0.90 ± 0.06	<0.0001	0.37	0.80
	MitoQ	0.83 ± 0.06	0.82 ± 0.03	0.87 ± 0.03			
HR (bpm)	Placebo	77 ± 17	95 ± 14	106 ± 17	<0.0001	0.90	0.97
	MitoQ	78 ± 23	96 ± 16	107 ± 16			
SBP (mmHg)	Placebo	111 ± 10	125 ± 13	136 ± 13	<0.0001	0.56	0.36
	MitoQ	111 ± 11	119 ± 10	131 ± 12			
DBP (mmHg)	Placebo	73 ± 13	71 ± 10	71 ± 11	0.55	0.82	0.58
	MitoQ	73 ± 10	71 ± 10	73 ± 10			
VE/*V*O_2_	Placebo	34.0 ± 3.61	28.0 ± 3.05	28.9 ± 3.54	<0.0001	0.95	0.70
	MitoQ	33.7 ± 4.00	28.5 ± 2.79	29.0 ± 2.85			
VE/VCO_2_	Placebo	40.1 ± 2.91	33.6 ± 3.17	32.1 ± 2.95	<0.0001	0.49	0.77
	MitoQ	40.6 ± 2.71	34.8 ± 2.57	33.1 ± 3.21			
O_2_ pulse (mL/beat)	Placebo	3.67 ± 1.02	7.66 ± 1.40	8.70 ± 1.29	<0.0001	0.94	0.56
	MitoQ	3.75 ± 1.18	7.64 ± 1.58	8.50 ± 1.60			
Fat Ox (%)	Placebo	47 ± 17	54 ± 16	32 ± 18	<0.0001	0.44	0.68
	MitoQ	55 ± 20	58 ± 12	38 ± 10			
CHO Ox (%)	Placebo	53 ± 17	46 ± 16	68 ± 18	<0.0001	0.29	0.34
	MitoQ	45 ± 20	43 ± 12	62 ± 10			

*Note*: Data are mean ± SD. *p*‐values represented for time, group, and interaction effects.

Abbreviations: CHO Ox, carbohydrate oxidation rates; DBP, diastolic blood pressure; Fat Ox, fat oxidation rates; HR, heart rate; O_2_ Pulse, oxygen pulse; SBP, systolic blood pressure; VCO_2_, volume of absolute carbon dioxide uptake; VE/VCO_2_, ventilatory equivalent for carbon dioxide; VE/*V*O_2_, ventilatory equivalent for oxygen.

### 2.3 Exercise protocol

One hour after supplement ingestion (Moreira et al., 1802; Park et al., 2020; Rossman et al., 2018), participants performed a graded exercise protocol on an electronically braked cycle ergometer (Lode Corival, Groningen, Netherlands). The protocol began with a 5‐min rest period seated on the ergometer followed by a 5‐min warm‐up at 30 W, and then workload progressively increased every third minute by 20 W until participants reached a respiratory exchange ratio (RER) of ≥1.0 (Dandanell et al., 2017). After a short break (2 min) during which participants remained seated on the ergometer, participants began an incremental exercise protocol (30 s at 30 W and 30 s at 100 W followed by 10 W increments every minute; average time to exhaustion of 4.6 ± 1.8 min and 5.0 ± 2.5 min for MitoQ and Placebo trials, respectively) until volitional exhaustion (Figure 1) (Dandanell et al., 2017). Expired air was analyzed via indirect calorimetry (COSMED Quark, Chicago, Illinois) using breath‐by‐breath analysis computed as a 30 s rolling average with outputs every 10 s to determine ventilation, volume of oxygen consumption, and volume of carbon dioxide production. *V*O_2max_ was achieved when the participant exhibited at least two of the following criteria: (1) plateau in *V*O_2max_ (≤2 mL kg min^−1^ or ≤ 150 mL with increasing workload), (2) a maximum heart rate (HR) (85% of age predicted maximum HR) (3) RER ≥1.1, (4) a rating of perceived exertion (RPE) above 17, or (5) a postexercise venous lactate concentration >8.0 mmol/L. Heart rate and ECG were continuously monitored throughout the exercise protocol. BP was measured during every third minute of the submaximal protocol and maximal protocols while RPE was measured every third minute during the submaximal protocol and every minute during the maximal protocol. Blood lactate was measured 1 min after the end of the maximal exercise portion (The Edge Lactate Monitoring System, ApexBio, Taiwan). Total exercise duration including baseline rest, warm‐up, exercise, and rest periods was ~30–45 min.

### 2.4 Evaluation of indirect calorimetry

Submaximal workload data were analyzed by averaging the last four readouts that averaged 10 s intervals between them representing the last minute of each submaximal workload. The crossover point was determined as the point at which 70% of the energy was carbohydrate based and 30% was lipid based (Brooks & Mercier, 1985). Absolute levels of fat and carbohydrate oxidation rates were determined via the Frayn equations and could then be converted to percent oxidized rates (Frayn, 1983). Tidal volume is represented using the body temperature, pressure, water vapor saturated (BTPS) denotation. Maximal workload data were analyzed by taking the highest absolute readout for each data point.

### 2.5 Statistical analysis

Data analysis was conducted using GraphPad Prism (version 9). A two‐way ANOVA analysis for Time × Trial was used for the submaximal workloads through 50 W (*n* = 9), as this was the highest workload all participants completed. Time represented the average value of individual variables during the last minute of each submaximal workload and Trial referred to either the Placebo or MitoQ trial. Paired *t*‐tests were used for individual points reached within the submaximal portion (substrate crossover wattage, substrate crossover time, absolute maximal fat oxidation, maximal fat oxidation wattage, time of maximal fat oxidation, time to RER of 1.0, absolute *V*O_2_ at RER of 1.0, relative *V*O_2_ at RER of 1.0, and RER of 1.0 as a percentage of *V*O_2max_). Paired t‐tests were also used for the maximal exercise portion. Partial eta squared (R2) was used to determine the effect size for the paired *t*‐tests. An alpha level of *p* < 0.05 was used to determine significance for all analyses. Data are represented as mean ± SD.

## 3 RESULTS

### 3.1 Submaximal exercise response

**FIGURE 2:**
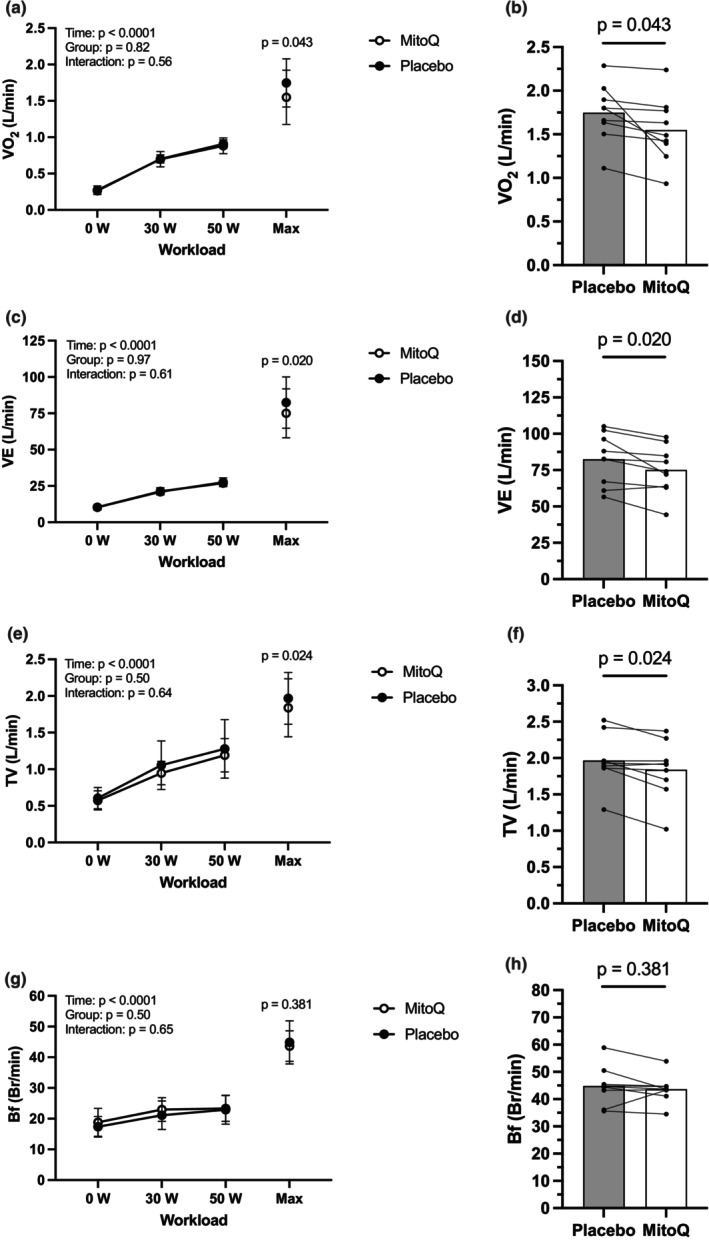
Metabolic and ventilatory variables' response to exercise bout. (a) Absolute levels of *V*O_2_ (L/min) response during the submaximal and maximal workloads. (b) *V*O_2_ (L/min) maximal exercise response with individual data points. (c) Absolute levels of VE (L/min) response during the submaximal and maximal workloads. (d) VE (L/min) maximal exercise response with individual data points. (e) Absolute levels of TV (L/min) response during the submaximal and maximal workloads. (f) TV (L/min) maximal exercise response with individual data points. (g) Absolute levels of Bf (Br/min) response during the submaximal and maximal workloads. (h) Bf (Br/min) maximal exercise response with individual data points. *V*O_2_, volume of oxygen uptake; VE, ventilation; TV, tidal volume; Bf, breathing frequency. Time, Group, and Interaction represent the statistical significance during the submaximal portion only. TV represented in the body temperature, pressure, water vapor saturated (BTPS) denotation. *n* = 9. *p < 0.05 versus Placebo. Data are mean ± SD.

There were no significant (*p* > 0.05) Time × Trial effects via the two‐way ANOVA through 50 W (*n* = 9) for submaximal absolute carbon dioxide uptake (VCO_2_), RER, heart rate, systolic BP, diastolic BP, ventilatory equivalent for oxygen (VE/*V*O_2_), ventilatory equivalent for carbon dioxide (VE/VCO_2_), oxygen pulse (O_2_ pulse), relative fat oxidation, and relative carbohydrate oxidation (Table 1). As expected, there was a difference (*p* < 0.0001) over time via the two‐way ANOVA through 50 W for submaximal absolute *V*CO_2_, RER, heart rate, systolic BP, diastolic BP, *V*E/*V*O_2_, *V*E/*V*CO_2_, O_2_ pulse, relative fat oxidation, and relative carbohydrate oxidation (Table 1). There was no difference (*p* > 0.05) for the Time × Trial effect but a difference (*p* < 0.0001) over time for absolute oxygen uptake (*V*O_2_), ventilation (VE), breathing frequency (Bf), and tidal volume (TV) via the two‐way ANOVA (Figure 2a,c,e,g). There was not a significant difference (*p* > 0.05) between trials for substrate crossover wattage, substrate crossover time, absolute maximal fat oxidation, maximal fat oxidation wattage, time of maximal fat oxidation, time to RER of 1.0, relative *V*O_2_ at RER of 1.0, absolute *V*O_2_ at RER of 1.0, and RER of 1.0 as a percentage of *V*O_2max_ (Table 2).

**TABLE 2:** Submaximal exercise responses during Placebo and MitoQ trials.

Variable	Placebo	MitoQ	*p*‐value	*R* ^2^
Substrate crossover (W)	59 ± 18	66 ± 13	0.35	0.111
Substrate crossover (min)	11.0 ± 2.0	11.1 ± 1.7	0.82	0.007
MFO (g/min)	0.28 ± 0.06	0.30 ± 0.07	0.39	0.095
MFO (W)	34.4 ± 8.8	26.7 ± 10.0	0.21	0.188
MFO (min)	7.7 ± 1.8	6.4 ± 2.3	0.35	0.110
RER of 1.0 (min)	18.1 ± 4.4	17.9 ± 3.2	0.77	0.011
*V*O_2_ at RER of 1.0 (mL/kg/min)	17.9 ± 5.4	16.7 ± 5.8	0.17	0.224
*V*O_2_ at RER of 1.0 (L/min)	1.32 ± 0.31	1.22 ± 0.32	0.18	0.213
RER of 1.0 (% of *V*O_2max_)	76 ± 13	80 ± 12	0.23	0.175

*Note*: Data are mean ± SD. *p* > 0.05 for all variables between trials.

Abbreviations: MFO, maximal fat oxidation; RER, respiratory exchange ratio; *V*O_2_, volume of oxygen uptake R^2^, effect size.

### 3.2 Maximal exercise responses

Relative *V*O_2max_ was higher (*p* = 0.03) in Placebo (23.5 ± 5.7 mL kg min^−1^) compared to MitoQ (21.0 ± 6.6 mL kg min^−1^) (Table 3). There were no significant differences (*p* > 0.05) between trials for maximal RER, maximal workload, maximal time to exhaustion, maximal lactate, maximal heart rate, maximal systolic and diastolic BP, maximal rate pressure product, maximal VE/*V*O_2_, maximal VE/VCO_2_, and maximal RPE (Table 3). Maximal oxygen pulse was higher (*p* = 0.03) in Placebo (10.96 ± 1.91 mL/beat) than MitoQ (9.86 ± 1.94 mL/beat) (Table 3). Maximal VCO_2_ was higher (*p* = 0.03) in Placebo (2.02 ± 0.42 L/min) compared to MitoQ (1.76 ± 0.41 L/min) (Table 3). Heart rate recovery (change in heart rate 1 min after exercise) was trending toward significance (*p* = 0.06) between Placebo (22.56 ± 10.53 Δbpm) and MitoQ (19.11 ± 11.25 Δbpm) trials (Table 3). Absolute *V*O_2max_ was higher (*p* = 0.04) in Placebo (1.75 ± 0.33 L/min) versus MitoQ (1.55 ± 0.37 L/min) (Figure 2b). Maximal ventilation was higher (*p* = 0.02) in Placebo (82.4 ± 17.7 L/min) compared to MitoQ (75.0 ± 16.8 L/min) (Figure 2d). Maximal tidal volume was higher (*p* = 0.02) in Placebo (1.97 ± 0.35 L/min) than MitoQ (1.83 ± 0.40 L/min) (Figure 2f). Maximal breathing frequency was not different (*p* > 0.05) between the Placebo (44.9 ± 7.0 Br/min) and MitoQ (43.6 ± 5.0 Br/min) trials (Figure 2h).

**TABLE 3:** Maximal exercise responses for Placebo and MitoQ trials.

Variable	Placebo	MitoQ	*p*‐value	*R* ^2^
*V*O_2_ * (mL/kg/min)	23.5 ± 5.7	21.0 ± 6.6	0.03	0.447
RER	1.17 ± 0.10	1.14 ± 0.06	0.21	0.188
Workload (W)	139 ± 26	135 ± 18	0.21	0.191
TTE (min)	5.0 ± 2.5	4.6 ± 1.8	0.19	0.203
Lactate (mmol/L)	10.6 ± 3.12	11.3 ± 3.92	0.48	0.062
HR (bpm)	169 ± 18	166 ± 17	0.11	0.279
SBP (mmHg)	172 ± 16	172 ± 20	0.90	0.002
DBP (mmHg)	70 ± 11	73 ± 9	0.15	0.242
RPP (mmHg × bpm)	29,007 ± 3849	28,438 ± 3787	0.56	0.045
VE/*V*O_2_	46.4 ± 6.7	49.7 ± 13.2	0.50	0.058
VE/VCO_2_	40.4 ± 3.3	44.7 ± 12.0	0.36	0.107
RPE	19.4 ± 0.5	19.6 ± 0.5	0.35	0.111
O_2_ pulse* (mL/beat)	11.0 ± 1.9	9.9 ± 2.0	0.03	0.456
VCO_2_ * (L/min)	2.02 ± 0.42	1.76 ± 0.40	0.03	0.459
HR Recovery** (Δ bpm)	22.6 ± 10.5	19.1 ± 11.3	0.06	0.380

*Note*: Data are mean ± SD.

Abbreviations: Bf, breathing frequency; DBP, diastolic blood pressure; HR, heart rate; HR Recovery, change in heart rate 1 min after exercise; O_2_ pulse, oxygen pulse; RER, respiratory exchange ratio; RPE, rating of perceived exertion; *R* ^2^, effect size; RPP, rate pressure product; SBP, systolic blood pressure; TTE, time to exhaustion; TV, tidal volume; VCO_2_, volume of carbon dioxide uptake; VE, ventilation; VE/VCO_2_, ventilatory equivalent for carbon dioxide; VE/*V*O_2_, ventilatory equivalent for oxygen; *V*O_2_, volume of oxygen uptake.

* *p* < 0.05 versus Placebo;

** *p* < 0.10 versus Placebo.

## 4 DISCUSSION

The current study assessed the acute effects of a mitochondrial targeting antioxidant on metabolic function during a single exercise bout in physically inactive females. Contrary to our hypothesis, MitoQ supplementation did not improve metabolic function during exercise. However, both maximal aerobic exercise capacity (i.e., *V*O_2max_) and maximal VE were both lower with acute MitoQ intake. This is the first study to analyze the acute effect of MitoQ on metabolic efficiency during submaximal and maximal exercise in inactive females and the first to show that MitoQ acutely impairs *V*O_2max_.

*V*O_2max_ is a whole‐body variable that integrates a multitude of physiological systems (Bassett Jr. & Howley, 2000). The current study implies that there are limited cardiovascular changes acutely caused by MitoQ in this population. While maximal HR and BP were not different between trials, O_2_ pulse, a surrogate for stroke volume, was lower during the MitoQ trial (Guazzi et al., 2017). Since maximal HR was not different, the change in O_2_ pulse can be attributed to the lower *V*O_2_. Thus, there were no apparent acute cardiovascular changes assessed in our study that explained the change in *V*O_2max_.

Interestingly, the lower *V*O_2max_ seen after consumption of MitoQ was primarily mediated by lower ventilatory function, specifically VE. Although VE does not directly represent the amount of oxygen being utilized by the working muscle, it is still highly related to both *V*O_2max_ and VCO_2max_ (Guazzi et al., 2017). VE is the product of tidal volume (TV) and breathing frequency (Bf) (Nicolò & Sacchetti, 2023; Tipton et al., 2017), and the lower TV with non‐different Bf during MitoQ insinuates that the change in VE was primarily augmented by TV. Both TV and Bf can be manipulated by neural inputs associated with the central command of the autonomic nervous system (Nicolò & Sacchetti, 2023; Prabhakar, 1985). MitoQ can surpass the blood brain barrier (McManus et al., 2011, 2014; Young & Franklin, 2019) and thus may act directly within the higher brain centers and influence central command regulation of VE. However, this is unlikely as the central command appears to regulate Bf to a much larger extent than TV (Nicolò & Sacchetti, 2023; Tipton et al., 2017). In contrast, metabolic factors such as arterial partial pressure of oxygen (PaO_2_), arterial partial pressure of carbon dioxide (PaCO_2_), and pH via the stimulation of both central and peripheral chemoreceptors (Nicolò & Sacchetti, 2023), which are controlled, in part, by a cell signaling cascade originating in the mitochondria (Donnelly & Carroll, 2005), regulate VE through TV (Nicolò et al., 2017, 2018; Nicolò & Sacchetti, 2023). Therefore, it is plausible that an altered cell signaling and function at the mitochondrial level could augment the ability of TV to maximally increase during exercise, and in turn decrease overall VE, thus lowering *V*O_2max_.

MitoQ has been shown to accumulate within the brain, skeletal muscle, and kidneys (Smith et al., 2003) and inhibit hypoxic pulmonary vasoconstriction in isolated mouse lungs (Pak et al., 2018). Pulmonary vasoconstriction is an acute response to hypoxia, which diverts blood away from under ventilated lung segments, thus promoting optimal perfusion. Exercise can also induce hypoxia and cause a change in redox signaling of the mitochondria within the pulmonary artery smooth muscle cells (PASMCs), resulting in pulmonary vasoconstriction (Dunham‐Snary et al., 2017). In turn, TV and VE are adjusted to respond accordingly to the stress imposed by alveolar hypoxia (Donnelly & Carroll, 2005; Dunham‐Snary et al., 2017). Therefore, acute MitoQ supplementation could alter the redox signaling capabilities by decreasing circulating mtROS levels outside of the optimal range to promote efficient function. Correspondingly, pulmonary vasoconstriction could be inhibited, thus inducing a decrease in the responsiveness of TV and VE, resulting in a decreased *V*O_2max_. Certainly, more work is needed to explore the impact of acute MitoQ supplementation on pulmonary function during exercise.

Our current findings have concluded that there was no acute impact of the supplement on submaximal exercise capacity or efficiency, yet there was a significant decrease in maximal exercise capacity. A study analyzing mtROS production and scavenging efficiency at high levels of exercise intensity concluded that maximal exercise performance is linked to the scavenging of antioxidant Exerkines released due to a multitude of redox signaling cascades caused by high mtROS production (Sawada et al., 2023). An exogenous antioxidant supplement could impact the efficiency of this cell signaling, which could have been interrupted due to a blunting of the rapid increase in mtROS and thus redox signaling seen with high levels of exercise intensity. However, mtROS generation is regulated to a much higher degree by the endogenous antioxidant system at lower levels of exercise intensity (Taherkhani et al., 2021). Therefore, the ability for an exogenous supplementation to blunt mtROS accumulation at this level of intensity could have been limited. Consequently, there could be a limited impact of exogenous antioxidants at the submaximal level, whereas there could be severe implications at maximal intensity, as seen in our study.

To our knowledge, the acute effect of MitoQ on exercise capacity has only been examined in patients with peripheral artery disease (PAD) (Park et al., 2020). In that study, 80 mg of MitoQ, equivalent dose as the present study, improved walking performance and time to claudication which was associated with higher levels of superoxide dismutase (SOD) and improved flow‐mediated dilation (Park et al., 2020). Unfortunately, differences in study design and populations make it difficult to directly compare results. Other studies have examined the chronic effect of MitoQ on exercise performance primarily in active populations. To start, 28 days of MitoQ supplementation (20 mg/day) improved cycling performance in middle‐aged recreationally trained male cyclists (44 ± 4 years) (Broome et al., 2021). The same group completed a follow‐up study analyzing the effects of 10 days (20 mg/day) of MitoQ supplementation on the skeletal muscle mitochondrial and antioxidant gene transcriptional response to high‐intensity interval training in recreationally active men (44 ± 7 years). Training‐induced increases in *V*O_2max_, 20 km cycle time trial performance, systemic markers of oxidative stress, and skeletal muscle oxidative stress markers were not influenced by MitoQ supplementation (Broome et al., 2022). While the current study did not have a direct measure of exercise performance other than *V*O_2max_, we did observe a reduction in *V*O_2max_ with no apparent change in peak workload with MitoQ, suggesting a potential influence on exercise efficiency. However, the *V*O_2_ to workrate slope was not different between trials, suggesting that the attainment of similar peak workload despite the systematic reduction in *V*O_2max_ can be attributed to the variability of anaerobic capacity between participants and the variability in the *V*O_2_ to workrate relationship between tests (Hansen et al., 1988; Poole & Richardson, 1997). Globally, the effect of MitoQ on exercise performance is not well understood and even less well studied is the impact of MitoQ on cardiopulmonary physiology.

### 4.1 Strengths and limitations

This study was the first to analyze a mitochondrial targeted antioxidants effect on metabolic function during exercise in physically inactive females. As a result, we were able to provide potential insight behind the mechanisms associated with redox signaling that are specific to optimal mitochondrial function, particularly in a very generalizable population group. Also, this study utilized a double‐blind placebo‐controlled design to limit the amount of bias by the participants and researchers. Additionally, this study provided context for further research involving the interaction between antioxidants and mtROS in promoting redox signaling in varying populations of differing physical activity status, cardiorespiratory fitness, sex, and ethnicity.

This study had some potential limitations that should be considered. To start, the entire population of the study was encompassed by non‐Hispanic White females. Racial and ethnic differences in mtROS generation and redox signaling is a relatively undiscovered area where more research is needed to elucidate potential ethnicity specific mechanisms. Additionally, the implications of age and sex differences are not fully understood related to this topic. Furthermore, our study utilized indirect calorimetry as its primary endpoint which may not be directly related to exercise performance. The use of additional measurements such as a pulse oximetry or arterial blood gas concentrations could have provided more evidence of changes in blood oxygen saturation previously eluded to in the discussion, which could support the proposed mechanisms being augmented by acute exogenous antioxidant supplementation. It should also be noted that there was not a direct measurement of circulating MitoQ or oxidative stress levels during either of the experimental trials. Finally, participants consisted of a mixed set of pre‐ and postmenopausal women which should be considered when interpreting the results.

## 5 CONCLUSION

In conclusion, acute MitoQ administration resulted in a lower TV and VE, resulting in a lower *V*O_2max_. Additionally, there was no effect of the supplement on substrate utilization or cardiovascular variables at the submaximal level. These findings help provide context behind the mechanisms that are reliant on optimal mtROS signaling and redox interactions. Notably, our findings are difficult to compare to other studies analyzing MitoQ due to either differences in dosage or population groups. Additionally, there is a lack of evidence to provide context behind the role of aging and sex differences related to this topic. Thus, further research should be done on varying populations in hopes of establishing the role exogenous antioxidant supplementation can play on this complex yet crucial cell signaling phenomenon.

## AUTHOR CONTRIBUTIONS

Ryan P. Hughes, Nicholas A. Carlini, and Matthew P. Harber conceived and designed research; Ryan P. Hughes and Matthew P. Harber performed experiments; Ryan P. Hughes and Matthew P. Harber analyzed data; Ryan P. Hughes, Nicholas A. Carlini, Bradley S. Fleenor, and Matthew P. Harber interpreted results of experiments; Ryan P. Hughes prepared figures; Ryan P. Hughes and Matthew P. Harber drafted manuscript; Ryan P. Hughes, Nicholas A. Carlini, Bradley S. Fleenor, and Matthew P. Harber edited and revised manuscript; approved final version of manuscript.

## FUNDING INFORMATION

Funding for this investigation was supported by the Clinical Exercise Physiology Program at Ball State University.

## CONFLICT OF INTEREST STATEMENT

No conflicts of interest, financial or otherwise, are declared by the authors.

## ETHICS STATEMENT

Each participant provided written informed consent as approved by the Ball State University Institutional Review Board.

## Data Availability

Data will be made available by the authors upon reasonable request.
